# Designing a novel hybrid healthcare teleconsultation network: a benchtop study of telepathology in Iran and a systematic review

**DOI:** 10.1186/s12911-020-01170-6

**Published:** 2020-08-12

**Authors:** Mohammad Mahdi Taghipour, Mohammad Mehdi Sepehri

**Affiliations:** The Laboratory for Healthcare Systems Optimization, Engineering, and Informatics, Faculty of Industrial and Systems Engineering, Tarbiat Modares University, Tehran, 1411713116 Iran

**Keywords:** Medical teleconsultation, Telepathology, Healthcare networks, Mathematical modeling, Resource optimization, Cost management

## Abstract

**Background:**

Growing demand for medical services has increased patient waiting time due to the limited number or unbalanced distribution of healthcare centers. Healthcare teleconsultation networks are one of the potentially powerful systems to overcome this problem. Medical pathology can hugely benefit from teleconsultation networks because having second opinions is precious for many cases; however, resource planning (i.e., assignment and distribution of pathology consultation requests) is challenging due to bulky medical images of patients. This results in high setup and operational costs. The aim of this study is to design an optimal teleconsultation network for pathology labs under the supervision of medical sciences universities in Tehran, Iran.

**Methods:**

To avoid the setup cost, we first propose a modified hybrid peer-to-peer (P2P) overlay architecture for our telepathology network, using Iran’s National Healthcare Information Network (SHAMS) as the underlying infrastructure. Then we apply optimization techniques to solve the request assignment and distribution problems in the network. Finally, we present a novel mathematical model with the objective of minimizing the variable operational costs of the system.

**Results:**

The efficiency of the proposed method was evaluated by a set of practical-sized network instances simulated based on the characteristics of SHAMS. The results show that the presented model and architecture can obtain optimal solutions for network instances up to 350 nodes, which covers our target network.

**Conclusions:**

We believe that the proposed method can be beneficial for designing large-scale medical teleconsultation networks by adjusting the constraints according to the rules and conditions of each country. Our findings showed that teleconsultation networks in countries with strong information technology (IT) infrastructures are under the influence of consultation fees, while in countries with weak IT infrastructure, the transmission costs are more critical. To the best of our knowledge, no research has so far addressed resource planning in medical teleconsultation networks using optimization techniques. Besides, the target network, i.e., pathology labs under the supervision of medical sciences universities in Tehran and the SHAMS network, are discussed for the first time in this work.

## Background

Long patient waiting time to receive medical services is one of the significant challenges in the healthcare systems of many countries [1]. Unfortunately, due to the increasing demand for these services as well as the limited number of healthcare resources, the average waiting time is increasing [2], causing the anxiety of the patients, delays in their diagnosis, and the possibility of deterioration of their conditions. All these lead to higher costs of treatment and less satisfaction of both patients and healthcare providers [3]. These problems being the case, many approaches have been proposed to reduce patient waiting time as well as possible. One of the most effective solutions is deploying teleconsultation networks [4]. A teleconsultation network is an online platform that connects specialists of a field through a secure network to share patient information [5]. Developing such platforms can have positive effects on the quality of care and the satisfaction of both patients and healthcare providers. It also would eliminate the problem of the geographically unbalanced distribution of medical specialists and facilities in many countries to some extent [6]. In addition, research findings have confirmed that a teleconsultation system could effectively reduce patient waiting time, decrease the overall cost of the healthcare system, and increase resource utilization [7–9].

One of the areas in which getting a second opinion or consultation is beneficial or even necessary in some cases is medical pathology [10]. Studies show that pathologists need to consult up to 20% of cancer cases in their routine work [11]. The consultation can be done through a telepathology system that employs telecommunications technology to exchange image-rich pathology data between different healthcare centers [12]. Designing such a network is challenging, considering the wide range of expertise in pathology and the large size of patient images. Furthermore, the risks, safety, legal implications, and liability of involved stakeholders should be taken into account [13]. However, the main obstructions to the widespread adoption of telepathology systems are the lack of suitable information technology (IT) infrastructure, the operation cost of the network, and the regulatory issues [14]. In this study, we mainly focus on the variable operational costs of the system.

Providing health services with the best possible quality and cost for patients and society is complex and challenging, because a variety of constraints regarding patients’ characteristics and needs, healthcare systems’ characteristics, limited resources, budgets, etc. should be taken into account. In order to find the best solution, rigorous and systematic approaches are required [15]. Optimization techniques are among the broadly utilized methodologies in modeling and solving quite complicated healthcare problems [16]. They can efficiently and systematically identify optimal solutions (best solutions) through optimizing a predefined objective function (Fig. 1).

In our problem, which is designing an optimal teleconsultation network through optimization techniques, we assume pathology labs and the healthcare centers with pathology departments are the nodes, and the communication links between them are the edges. Nodes can be both requesting nodes (having consultation requests) and consulting nodes (giving consultation to the others). For satisfying each consultation request, there are two types of costs involved:

The first type is the cost of request transmission from the requesting nodes to the consulting nodes. In our network, a consultation request may pass through several links and transit the nodes to get to its destination. So, the transmission cost for each consultation request is the cost of the links, which transfer it. By proposing a hybrid network architecture, there is no need to set up any connections; instead, we lease the links. The leasing cost of each link mainly depends on the reserved capacity and its quality of service.

The second type is the consultation fee of the consulting nodes for responding to the requests. The consultation fee of each node depends on different factors such as the skill and expertise level of its specialists, geographical location, available facilities, number of specialists, crowdedness, etc.

Additionally, increasing demand for exchanging electronic health records and medical images has made the growth of healthcare information networks indispensable. Many countries have built [17–19] or are developing [20–22] their national healthcare information network. Such a system provides a common platform for information exchange across diverse healthcare entities. It can be an appropriate underlying network that enables us to create overlay service networks on top of it. An overlay network is a virtual topology that is built on the top of another network and has a high potential for cost savings [23]. Nodes in this network are purposefully selected amongst all the nodes of the underlying systems. Besides, links can be either virtual or physical; a physical link is a direct route between two nodes in the underlying network while a virtual link is a route that may pass many physical nodes that are not included in the overlay topology (Fig. 2).

Peer-to-peer (P2P) systems are examples of overlay networks for large-scale services over public networks [23]. They are known as promising solutions, which overcome many challenges in distributed and collaborative networks [24]. In such systems, each node can act both as a server (providing a service or information) and a client (receiving a service or information). They are scalable and resistant to a single point of failure. These features make the p2p architecture suitable for a teleconsultation network. While most studies in this field have focused on the content distribution problem, there is another challenge in assigning the requests to the consulting nodes. In the present work, these two subproblems should be solved simultaneously.

For the challenges mentioned above, there are two key motivations:

1) To decrease patient waiting times for accessing a more accurate diagnosis by designing an effective teleconsultation network. Most of the existing works in this field have focused on the implementation techniques for teleconsultation networks, while a few studies have worked on the effective designing of the architecture of these systems. In this work, we propose an architecture of a P2P overlay network that can be adopted by any existing underlying networks such as national healthcare information networks. Due to the existence of Iran’s National Healthcare Information Network (SHAMS), the idea of deploying SHAMS as an underlying network for telepathology services motivated us to create an efficient overlay telepathology network.

2) Optimizing the performance of the network through minimizing the system’s variable costs, including the consultation fees and transmission costs. The problem addressed in this study has formed based on many interviews with Iranian pathologists discussing their challenges. To the best of our knowledge, this is the first work that proposes such a comprehensive mathematical model for a medical teleconsultation network.

Our contributions include precise descriptions of the main concepts of the proposed P2P overlay teleconsultation network and a methodology to design its architecture. Additionally, we formulate a novel Integer Linear Programming (ILP) optimization model for the problem in order to optimize the request assignment and the content distribution at the same time. We also show that the mentioned problem is NP-hard.

Our target network is the set of medical pathology labs under the supervision of medical sciences universities in Tehran. So, in order to have a valid underlying network, we simulated the characteristics of the SHAMS network. The results show that the proposed model is capable of solving all real-sized instances optimally. Furthermore, we conducted numerical experiments to evaluate the sensitivity of the model to various parameters.

## Methods

### Literature review

Since our subject falls into an interdisciplinary area, the literature is multifaceted. There are many papers on this subject but from different perspectives. Therefore, we provide an overview of the academic publications mainly in two areas: 1) papers/reports of telepathology systems, and 2) optimization methods for request assignment and content distribution in P2P and overlay networks.

For this purpose, we reviewed the papers in Google Scholar published between 2000 and 2019. For each area, first, we started searching in Google Scholar with a few general keywords. Then with the help of studies that seemed to be related to our work, we extracted our accurate keywords which were: medical consultation networks, teleconsultation, telepathology, P2P healthcare networks, peer to peer networks, overlay networks, resource planning, task assignment, content distribution, routing, mathematical model and optimization.

After that, since full-text research in Google Scholar returns thousands of irrelevant records, we performed smart searches using the keywords “intitle” and “intext”. The search strategy is provided in Additional file 10

Totally, we found 812 records. Then in each of the searches, we reviewed the articles very briefly (mostly as titles) and eliminated irrelevant articles. Consequently, 637 citations were extracted in the Identification step. After adjusting for duplicates, 329 citations remained. Then, after screening the abstracts, 245 studies were excluded. The full-text articles of the remaining 84 studies were examined for eligibility, and 31 studies were selected for inclusion in the review. More details are provided in the PRISMA flow diagram (Fig. 3).

In the first area, most of the related works have emphasized on the importance and benefits of teleconsultation networks by reporting the best practices, while few researchers have addressed designing the architecture of these systems. As far as we know, no one has presented any cost or resource optimization model for such networks so far. On the other hand, in the second area, there are some similarities between the characteristics of our problem and others in the field of P2P overlay networks; again, we could not find any work addressed a simultaneous request assignment and content distribution problem fitting to our constraints.

#### Papers/reports of Telepathology systems

Weinstein et al. [25] reviewed the concepts and methods for the implementation of telepathology. They categorized telepathology practice models and claimed that healthcare systems could benefit from economies of scale when telepathology services become available in public networks such as the Internet. Teleconsultation and clinical decision-making systems are also reviewed in [26]. They classified the consultation requests into four categories: 1) Referral, 2) Diagnosis, 3) Treatment, and 4) Education. Based on their report, teleconsultation between two levels of experts or centers was more effective for referrals (mostly reducing referrals) and treatment.

Moreover, there are extensive reports on the implementation of telepathology systems and their performances in different countries such as the United States [27, 28], Japan [29, 30], Germany [31, 32], Canada [33, 34], the Netherlands [35, 36], Africa [37, 38] and Hungary [39, 40]. Similarly, [41] showed the results of the two-year implementation and experiences of a nationwide telepathology consultation program in China. These researches verified that telepathology could solve the problem of the unbalanced geographical distribution of pathology resources and could play an essential role in improving the pathology diagnosis.

Some studies, like [42], investigated technological solutions to overcome the challenges associated with the distribution of large images across a network or the Internet. These challenges especially come from pathology practices. Telepathology systems do not necessarily require specialists to be available at the same time. Therefore, telepathology platforms use an asynchronous (store-and-forward) method for data transmission. This method removes many difficulties, including scheduling a meeting time for specialists who have hectic work schedules and are apart geographically. However, the network architecture of these systems has a vital role in their overall performance [43].

McCarthy et al. [44] categorized the architectures of existing healthcare information networks into three general models: 1) Centralized model, 2) Decentralized model, and 3) Hybrid model. More details on these three models will be given in the Methods section. McCarthy et al. [44] indicated that healthcare organizations choose between the mentioned models based on their local needs, objectives, and policy, legal and market conditions. As an example, [45] proposed a decentralized architecture for pathology networks due to the large storage size of pathology slide images.

These papers were just a few examples showing that the teleconsultation networks are getting much attention in recent years; however, they still have some unsolved challenges. We could not find any papers that present any resource optimization models. The review of all the best practices is beyond the scope of this study.

#### Request assignment and content distribution problems in P2P overlay networks

The literature of P2P overlay networks mostly tends to focus on the optimization approaches for topology design, resource assignments, and content distributions in such systems. As discussed before, in our problem, we have some requesting nodes, which have consultation requests (containing patient medical images) that should be transferred to the consulting nodes. The assignment and distribution of the requests should be based on the minimization of the transmission cost and the consultation fee. Therefore, there are two subproblems at the same time. Studies in this field usually focus on just one of the mentioned subproblems, assuming that the other one has been determined. In addition, we have some constraints such as the capacity of links between the nodes and the minimum and maximum capacity of each node for responding to the requests.

Killian et al. [46] described the content distribution problem in overlay networks. They formulated the problem, assuming that all content is in the form of unit-sized tokens. Distribution of tokens proceeds as a sequence of time steps. Similar to our problem, they assumed that the capacity of every overlay link is limited. They proved that the problem is NP-hard. Based on their time sequence idea, Chmaj et al. [47] proposed an optimization model for P2P computing systems aiming at minimizing the transmission and processing costs. They first assumed that in each iteration (time slot), the data would be transferred between the nodes in block form. Then they added an extra decision variable for assignment of the blocks to each node for the processing. Considering the processing cost as the consultation fee, our problem shares some similarities with the above work. However, the difference between the problems is that in their model, each processed block should be distributed among all the nodes, while we only send each request to a certain predetermined number of consulting nodes. Also, we have different request types; for each type only a subset of nodes can respond, and there are additional constraints on the minimum and maximum responding capacity for each node.

Other related works in this field have only focused on the content distribution, i.e., routing problem. For instance, Rai et al. [48] described a network in which the underlying paths are predetermined and unknown to the overlay network. Then they presented an optimal dynamic routing algorithm based on the congestion of the links in the underlying network with predetermined destinations. Likewise, we have capacitated links in the underlying network and we need to develop a content distribution model, but the decision should be made based on the end node cost (consultation fee) as well.

Moreover, Yang et al. [49] studied restricted overlay routing. In order to control the overall performance of the system, they shared only a part of the information of the overlay network with each node. So, the nodes should specify their end-to-end routes according to their demands and limited knowledge of the overlay network. We have a similar situation in our problem when there are different request types; again the destinations for the requests are not determined beforehand. Both of the mentioned studies evaluated their results through instances generated by simulations under various topologies and parameters.

Applying a different approach, Maiti et al. [50] designed a system for remote access laboratories that enables P2P experimental design and sharing. They proposed a generic architecture that could be applied to any distributed P2P network control systems over the Internet. Besides, they presented a framework distributed remote control regarding remote access laboratories.

Carbajo and Mc [51] presented an architecture for scalable data transmission in wireless sensor networks in which the content is impartially transferred between client and server nodes in an overlay manner. The system was assessed under different network conditions and scenarios via simulations and proved to be reliable and scalable, enjoying a fair performance for content distribution. For more information on P2P overlay networks, refer to [23, 52].

In conclusion of this part, it is clear from the papers reviewed that healthcare teleconsultation is much immersed and widely practiced, and there are many outstanding works in both areas of “implementation of teleconsultation networks” and “P2P overlay networks”. However, there is a research gap in the modeling of teleconsultation networks in order to obtain optimal solutions for resource planning and cost management. To the best of our knowledge, this problem has not appeared in any previous work so far. Also, this is the first time that our target network is investigated in the literature from a network optimization perspective.

In the rest of this section, we formulate a novel ILP model for a teleconsultation network. As discussed previously, developing such a network is one of the most effective ways of speeding up the diagnosis and reducing the overall costs of treatment. So, we focus on characterizing the problem, especially in pathology field, without loss of generality. The reason behind this choice is that in pathology, the size of the patient’s slide images is large, and it is a real challenge for such networks. Besides, the ownership right of the images should be protected for each center or lab, which created them by not duplicating the images in the destination (consulting node) after the response. It is known that ILP modeling is a research approach extensively exploited in optimization of different types of systems like computer networks [53].

### Assumptions

In order to develop a mathematical model for such networks that guarantees a certain quality of service with the lowest cost of operation, the structure of the underlying network (e.g., a national health network or any other public network) should be known. Also the location of the pathology centers in the network, the average number of consultation requests, and the consultation fees of each center are required.

Knowing this information, the objective function is to minimize the variable operational costs of the network: the request transmission costs and the consultation fees. For the sake of simplicity, we have eliminated the fixed costs and just focused on two variable costs associated with transmissions and consultation fees.

A weighted undirected graph represents the underlying network of the model including *N* nodes indexed by *n* = 1, …, *N*. We categorized the consultation requests into different types indexed by *t* = 1, …, *T* based on the pathology subspecialties they needed. The other assumptions of the problem are as follows:

- In this network, all overlay nodes are pathology labs, or healthcare centers with a pathology department.
- All nodes are provided with the required hardware and software to capture, store, view, and share the images such as PACS.
- The network’s edges are telecommunication links between the nodes. These links can be dedicated connections through an intranet or virtual connection over an underlying network.
- Nodes can be both requesting nodes and consulting nodes. However, each node cannot respond to its requests.
- We categorized the consultation requests into different types based on the subspecialties they needed.
- In each node, there are pathologists with different subspecialties. Therefore, each node can only answer to the requests with related subspecialty.
- Each requesting node can indicate a list of consulting nodes that are qualified to respond to its requests with attention to all aspects, including their knowledge, expertise, and even skill level. In addition, its requests can only be answered by only those nodes.
- The consultation fee for each request type is different.
- Since it is a cooperative network, we oblige each node to answer a minimum number of requests of each type. Also, in order to preserve the quality of consultation service, the nodes are not allowed to accept requests more than a maximum number defined for each type. These two numbers are predetermined for each node according to many factors, including its capabilities and specialty level.
- For some requests, more than one, e.g., two or three consultations from different consulting nodes are required.
- If, in a node, more than one pathologist responds to a consultation request, the node’s final answer to that request is the consensus of all opinions.
- The network flow is a sequence of requests passing through the network nodes and links to get from the requesting nodes to the consulting nodes. The amount of flow goes into a node from each source equals to what goes out, unless the node accepts some of the requests.
- Each request may go through some transit nodes to get to its destination.
- The transmission rate (or the capacity of the links) is defined as the maximum total number of requests passing through each link in both directions in the time period of the problem.
- The ownership right of each digital pathology image belongs to the laboratory that produces it, according to the current situation of digital pathology in Iran.
- To protect the ownership rights of the slide images, we do not duplicate the requests in different nodes for further uses; rather we delete them at the destination after the consulting nodes respond to them.
- Since we use an overlay architecture, the cost of establishing the links is zero, but the operation cost of them is considered as the cost of request transmission.
- The time horizon is assumed to be one working day.

### Network architecture

Here, we concisely describe the proposed architecture of our network. The characteristics of this network, (being an overlay network and the roles of each node as both client and server) seem to match the functionalities of P2P systems. Additionally, P2P systems have some desirable features like scalability and autonomy that suit our network.

As mentioned before, healthcare information networks are categorized into centralized, decentralized, and hybrid models. A centralized model refers to a network in which all data are stored in a central node (repository) while the other nodes can retrieve them according to the network’s policies and procedures. Because all data are housed in a single node, it is very straight and fast to access them; however, it has the problem of a single point of failure. On the other hand, in a decentralized model, each source node is responsible for its data and has to maintain it by itself. Any query for specific data should be made only to the source node of the data. Finding the location of the source nodes in this model is challenging. Finally, hybrid models are any combination of centralized and decentralized models in which there is a data finder (locator) service for the nodes to access the source nodes of the required data. Data in such a model can be aggregated in a central node or stored in distributed nodes. Although all existing systems can be classified into one of the mentioned categories, there are differences between the systems and their implementations. Researchers may choose the same name for their architecture, even when they deploy different configurations [44].

In light of these notes, the proposed architecture is described as follows. Since pathology images have large storage sizes, transferring them to a central node requires much time and high bandwidth. Besides, it would be costly, and the central node should have a huge data repository, which brings another challenging issue. Therefore, we believe that our network must have a distributed architecture for the storage of pathology slide images. In this way, we will be able to protect the ownership right of the images with less difficulty by giving the assess control of each node’s data to itself.

Furthermore, in our network, there is an organizer node, aware of all the network information, to assign and route the requests to the consulting nodes. Then the requests will be delivered to their destinations through the P2P approach. The organizer node does not collect the contents of the requests; instead, it gets updated about the type and the location of them. It also monitors the responding capacity of the consulting nodes as well as the capacity of the links.

In our hybrid P2P overlay architecture, all the nodes have equal rights and are responsible for their data. Besides, we have a high level of collaboration and scalability. The architecture of our network is depicted in Fig. 4.

### Model

In this part, we formulate the described network based on the proposed architecture. The model optimizes the operating cost of the network, including two key factors: the consultation fee of each node and the transmission cost of each request. The parameters, decision variables, and ILP model of the problem are as follows:Taba

*Parameters*

**Table:** 

*c*_*ij*_	The cost of request transmission from nodes *i* to node *j*
${a}_i^t$	The consultation fee of node *i* for responding to each request of type *t*
${h}_i^t$	The maximum number of consultation requests of type *t* that node *i* can respond
${l}_i^t$	The minimum number of consultation requests of type *t* that node *i* should respond
${r}_i^t$	The number of consultation requests of type *t* that node *i* has per day
*u*_*ij*_	The maximum capacity of the link between node *i* and node *j* in both directions
${x}_{ik}^t$	$$ \left\{\begin{array}{c}1\ \mathrm{if}\ \mathrm{node}\ i\ \mathrm{is}\ \mathrm{allowed}\ \mathrm{to}\ \mathrm{respond}\ \mathrm{to}\ \mathrm{the}\ \mathrm{requests}\ \mathrm{of}\ \mathrm{type}\ t\ \mathrm{from}\ \mathrm{node}\ k\ \\ {}\\ {}0\ \mathrm{Otherwise}\ \end{array}\ \right. $$
*m*^*t*^	The number of consultations needed per request for the requests of type *t*

*Variables*

**Table:** 

${F}_{ijk}^t$	The number of type *t* requests belonging to node *k* and passing through the link between node *i* and node *j*
${P}_{ik}^t$	The number of type *t* requests belonging to node *k* that node *i* responds to

As described, ${F}_{ijk}^t$ is a flow variable that indicates the path of the requests and ${P}_{ik}^t$ assigns the requests to the consulting nodes.

*Objective*

1 $$ \mathit{\operatorname{Min}}\ \left\{\sum \limits_{t\in T}\ \right.\sum \limits_{i\in N}\ \sum \limits_{j\in N}\ \sum \limits_{k\in N}{F}_{ij k}^t\ {c}_{ij}+\sum \limits_{t\in T}\ \sum \limits_{i\in N}\ \left.\sum \limits_{k\in N}{P}_{ik}^t{a}_i^t\ \right\} $$

Subject to

2 $$ {P}_{ii}^t=0,\forall i\in N,\forall t\in T $$

3 $$ {F}_{iij}^t=0,\forall i,j\in N,\forall t\in T $$

4 $$ {P}_{ik}^t\le {x}_{ik}^t.{r}_k^t,\forall i,k\in N,\forall t\in T $$

5 $$ \sum \limits_{j\in N}{F}_{jik}^t=\sum \limits_{j\in N}{F}_{ijk}^t+{P}_{ik}^t,\forall i,k\in N,\forall t\in T $$

6 $$ \sum \limits_{k\in N}{P}_{ik}^t\le {h}_i^t,\forall i\in N,\forall t\in T $$

7 $$ \sum \limits_{k\in N}{P}_{ik}^t\ge {l}_i^t,\forall i\in N,\forall t\in T $$

8 $$ \sum \limits_{t\in T}\ \sum \limits_{k\in N}\left({F}_{ij k}^t+{F}_{jik}^t\right)\le {u}_{ij},\forall i,j\in N $$

9 $$ \sum \limits_{j\in N\ }{F}_{iji}^t={m}^t{r}_i^t,\forall t\in T,\forall i\in N $$

10 $$ \sum \limits_{i\in N}{P}_{ik}^t={m}^t{r}_k^t,\forall t\in T,\forall k\in N $$

11 $$ {F}_{ijk}^t,{P}_{ik}^t\ge 0\kern0.28em and\kern0.28em \mathrm{integer},\forall t\in T,\forall i,j,k\in N $$

Where the objective function (1) focuses on minimizing the variable cost of the network by joint consideration of the distribution and assignment costs. Constraint (2) ensures that each node cannot respond to its requests. Constraint (3) imposes that there is no flow between a node and itself. Constraint (4) controls which nodes are allowed to respond to the requests of type *t* of node *k*. It is defined for the situation where a consulting node may not have the eligibility to provide consultation service to some request types, or the requesting node does not want a specific consulting node to respond to its requests. Also it ensures that the total number of requests of type *t* that a consulting node responds from a specific node should not exceed the total number of requests of type *t* of that node. Constraint (5) guarantees that the incoming flow to each node is equal to the outgoing flow plus the number of requests that the node has accepted to respond. Based on Constraint (6), each node cannot exceed its maximum acceptance capacity for each request type in order to preserve the quality of consultation service. Constraint (7) also denotes the minimum number of requests that must be answered by each participant node in the system. Constraint (8) controls that the capacity of each link should not be violated by the total amount of flows passing through it in both directions. Constraint (9) guarantees that all the requests of each node should be distributed through the network. Constraint (10) ensures that all requests of all nodes should be responded to. Finally, Constraint (11) indicates that the decision variables are non-negative integers.

The ILP model (1–11) can be reduced to the Integer Multi-Commodity Flow (IMCF) problem, which is proved to be NP-hard [54].

## Results and discussion

In this section, we evaluate the performance and applicability of the proposed model, applying numerical test problems simulated by the topology model benchmark of public networks presented in [55]. For our experimental study, we looked over the characteristics of the SHAMS network. SHAMS is the telecommunication infrastructure of Iran’s Ministry of Health and Medical Education created independently of the Internet for exchanging information and data between the healthcare centers. It has been developed since October 2013 and covers more than 9500 healthcare centers. We also collected information about the pathology labs under the supervision of medical sciences universities in Tehran to get more realistic ideas about our network instances. Based on this information, we adjusted the parameters of our topology function. The parameters of each test problem are presented in Table 1. In this table, *α* controls the connectivity of the graphs, *β* has a direct effect on the number of edges, and *ε* adjusts the average node degree (Avg Deg in the table).

**Table 1:** Underlying network topology classes

N^a^	Avg Deg^b^	α	β	ε	N	Avg Deg	α	β	ε	N	Avg Deg	α	β	ε
50	4	0.65	0.15	3.12	200	4	0.63	0.13	3.04	300	4	0.61	0.11	3.00
	8	0.94	0.15	3.12		8	0.93	0.13	3.04		8	0.91	0.11	3.00
	12	0.97	0.14	3.26		12	0.98	0.14	3.32		12	0.98	0.15	3.40
	16	1.12	0.17	3.12		16	1.12	0.17	3.08		16	1.15	0.18	3.00
100	4	0.64	0.13	3.09	250	4	0.63	0.12	3.02	325	4	0.60	0.11	3.00
	8	0.94	0.13	3.09		8	0.92	0.12	3.02		8	0.90	0.11	3.00
	12	0.98	0.14	3.26		12	0.98	0.14	3.32		12	1.00	0.15	3.45
	16	1.12	0.17	3.09		16	1.15	0.17	3.40		16	1.15	0.18	3.00
150	4	0.64	0.13	3.07	275	4	0.62	0.12	3.01	350	4	0.60	0.10	3.00
	8	0.93	0.13	3.07		8	0.92	0.12	3.01		8	0.90	0.10	3.00
	12	0.98	0.14	3.30		12	0.98	0.14	3.40		12	1.00	0.15	3.50
	16	1.12	0.17	3.07		16	1.15	0.17	3.02		16	1.20	0.18	3.00

^a^N: Network Nodes, ^b^Avg Deg: Average Node Degree

We defined 36 classes for the underlying network (nine levels for the number of nodes and four levels for the average node degree). For each class, we generated 10 test problems based on the model parameters, as mentioned before. These test problems are generated randomly based on uniform distributions within the stated intervals in Table 2. Finally, to obtain the results for each class, we ran the model for ten test problems of that class and computed the average results. All information for the underlying network topology and the parameters of the model were collected through the interview with experts.

**Table 2:** Intervals for the model parameters in the test problems

Parameter	Range
${\mathrm{a}}_{\mathrm{i}}^{\mathrm{t}}$	*~U*(15, 34)
c_ij_	*~U*(4, 12)
u_ij_	*~U*(0, 45)
${\mathrm{r}}_{\mathrm{i}}^{\mathrm{t}}$	*~U*(30, 54)
${\mathrm{h}}_{\mathrm{i}}^{\mathrm{t}}$	*~U*(30, 59)
${\mathrm{l}}_{\mathrm{i}}^{\mathrm{t}}$	*~U*(5, 14)

The proposed model was implemented in the ILOG CPLEX optimization studio environment, and the datasets were generated using the Matlab software. All tests were executed on a machine with Intel Xenon(R) CPU E5–2620 2.40GHz (2 Processors) and 16 GB of RAM. For instance, a solution for an example network of 10 nodes is visualized in Fig. 5.

### Effect of the number of nodes

In order to analyze the effect of network size on the performance of the model, we used nine classes of the underlying network topology with the average node degree of 4, and in each class, we ran the model on ten test problems. We report the average of the results and computational times in Table 3. As shown, the proposed model can achieve the optimal solutions for the instances up to 350 nodes on the deployed machine. This number of nodes covers the number of pathology labs in our case study. Also, as we expected, the variable operational costs of the system increased according to the size of the network.

**Table 3:** Effect of the number of nodes on the performance of the model

No.	Network nodes	Computational time (Secs)	Consultation cost	Transmission cost
1	50	1.65	50,540	16,259
2	100	7.92	103,067	31,039
3	150	19.69	162,251	49,545
4	200	51.87	204,702	63,895
5	250	91.29	247,532	83,678
6	275	120.21	277,292	85,492
7	300	172.44	303,149	95,089
8	325	241.97	335,138	104,507
9	350	350.01	360,547	112,430

Furthermore, the transmission costs represent nearly 30% of the costs; this ratio does not change much when the number of nodes goes up. These results show that the consultation fees are almost twice as influential as the transmission costs in the management of the system. However, as we will illustrate that later this situation may change when the underlying network capacity changes.

Besides, as stated previously, the problem is NP-hard, and our experiments show that the computational time increases non-linearly as the network size gets larger (Fig. 6).

### Effect of the average node degree

The average node degree represents the degree of freedom in choosing the paths for the requests in a network. So, we used all the 36 classes of Table 1 and ran the model on ten test problems for each class. The average of the results is provided in Table 4. As the results show, the computational time rises when the average node degree increases. This effect may come from the fact that by increasing the average node degree in the network, we will have more links (the bigger solution space) to search for optimal paths. However, the effect of the average node degree is less intense on the computational time when compared to the effect of increasing the number of nodes (Fig. 7).

**Table 4:** Computational times for different average node degrees

No.	Network nodes	Computational times for Avg. Deg. of 4	Computational times for Avg. Deg. of 8	Computational times for Avg. Deg. of 12	Computational times for Avg. Deg. of 16
1	50	1.65	2.42	3.23	3.96
2	100	7.92	9.36	11.87	14.04
3	150	19.69	23.95	27.94	30.56
4	200	51.87	53.76	58.09	63.45
5	250	91.29	95.72	99.98	109.78
6	275	120.21	132.05	135.08	148.68
7	300	172.44	180.34	184.16	196.01
8	325	241.97	261.46	285.83	311.60
9	350	350.01	374.64	409.21	443.73

Interestingly, no strong relation was observed between the costs and the average node degree in the results. In other words, when the average node degree increased, the variable operational costs of the system did not change significantly.

### Effect of the capacity of links

As mentioned, in the proposed model, we are trying to assign and route the requests to the consulting nodes simultaneously, aiming at minimization of the variable operational costs. In the routing process, two factors may affect the optimal path: transmission cost and capacity of each link. In this part, we analyze the sensitivity of the model to the changes in the links’ capacities. For this purpose, we deployed a complete graph as the underlying network to evaluate the model more comprehensively. Because the processing memory of the machine was limited, we ran the model on the test problems with the number of nodes from 50 to 200 and the average node degree of 4. In order to investigate the effect of the capacity of links, we defined two scenarios in this part.

In the first scenario, we compared the results of the model on the test problems with capacitated and uncapacitated links. This being the case, we generated ten test problems with capacitated links and ten test problems with uncapacitated links, all of them with 50 to 200 nodes and the average node degree of 4. Other parameters were generated randomly within the intervals given in Table 2.

According to Fig. 8, the computational time increases for the uncapacitated test problems to the extent that this increase reaches about 40% at 200 nodes. Also, like the previous part, no substantial difference was found between the costs of capacitated and uncapacitated networks.

In the second scenario, we decreased the average capacity of the links from 70 to 2.5 to evaluate the results. The computational time and the transmission cost difference ratio between the results for the average capacity of 70 and the results for the other average capacities are depicted in Fig. 9. It is noticeable from the results that decreasing the capacity of links directly increases both the computational time and the transmission cost with different trends. It is very likely that by decreasing the capacity of links, finding feasible solutions may become hard, and the computational time will increase. In addition, the observed increase in the transmission cost is due to longer paths (or more transit nodes) that each request should pass to get to the destination. It means that when the underlying network is weak in capacity, the transmission cost becomes a more dominant factor for assigning the consultation requests to the consulting nodes. In other words, the implementation of the teleconsultation networks in countries with strong information technology (IT) infrastructures is under the influence of the physicians’ fees while in other countries, the transmission cost is more critical.

## Conclusions

We studied the medical teleconsultation networks having high potentials to decrease the costs of related medical services and patient waiting times. We also addressed the research gap in the modeling of such networks in order to obtain optimal solutions for resource planning and cost management. Then we described a hybrid P2P overlay architecture for such a network and formulated a novel ILP model that optimizes request assignment and content distribution simultaneously. The set of pathology labs under the supervision of medical sciences universities in Tehran and the SHAMS Network were studied as the case study. Finally, we did comprehensive tests on 580 test problems in 36 classes of the underlying network topology. The computational results showed that our proposed model could achieve optimal solutions for instances up to 350 nodes, which covers the case study. The findings further showed that in countries with better ICT infrastructure, the consultation fee has a stronger effect on the costs of such networks, while in other countries, the transmission cost is a challenging issue. We believe that our method and the results presented in this work can be valuable for designing nationwide medical teleconsultation networks. As far as we know, this problem has not so far been addressed and formulated in any research community. Also, our case study is discussed for the first time in this work.

This research has a fundamental role in designing healthcare teleconsultation systems. Although we made some simplifications in the proposed ILP model of these networks compared to real systems, even the simplified model is complicated and computationally challenging according to the experiments. For future work, we hope to implement this network by persuading the stakeholders and administrations of our case study. In addition, we plan to make the model more realistic by considering the following aspects: uncertainty in demands, employing links with modular capacities, and algorithms for a nationwide scale of the problem.

## Supplementary information

**Additional file 1: Fig. 1** The framework of the proposed methodology

**Additional file 2: Fig. 2** An overlay architecture for healthcare teleconsultation networks.

**Additional file 3: Fig. 3** The PRISMA flow diagram of the literature search

**Additional file 4: Fig. 4** The proposed hybrid architecture for healthcare teleconsultation networks

**Additional file 5: Fig. 5** A visualized solution for a network of 10 nodes, nodes and links with a bigger flow have a bigger size

**Additional file 6: Fig. 6** The computational time versus the number of nodes

**Additional file 7: Fig. 7** The computational time versus the average node degree

**Additional file 8: Fig. 8** The computational time for the capacitated and uncapacitated networks

**Additional file 9: Fig. 9** Average of the computational time and the transmission cost differences versus the capacity of the links

**Additional file 10: Appendix I**. The Search Strategy

## Data Availability

All the datasets, topology classes, and codes used and analyzed during the current study are available from the corresponding author on reasonable request.

## Abbreviations

P2P: Peer-to-Peer
IT: Information Technology
ILP: Integer Linear Programming
Avg Deg: Average Node Degree
