# Supplementary figures and images for: Designing a novel hybrid healthcare teleconsultation network: a benchtop study of telepathology in Iran and a systematic review
Source: BMC Med Inform Decis Mak. 2020 Aug 12;20:186. doi: 10.1186/s12911-020-01170-6 (PMC7477836; doi:10.1186/s12911-020-01170-6)

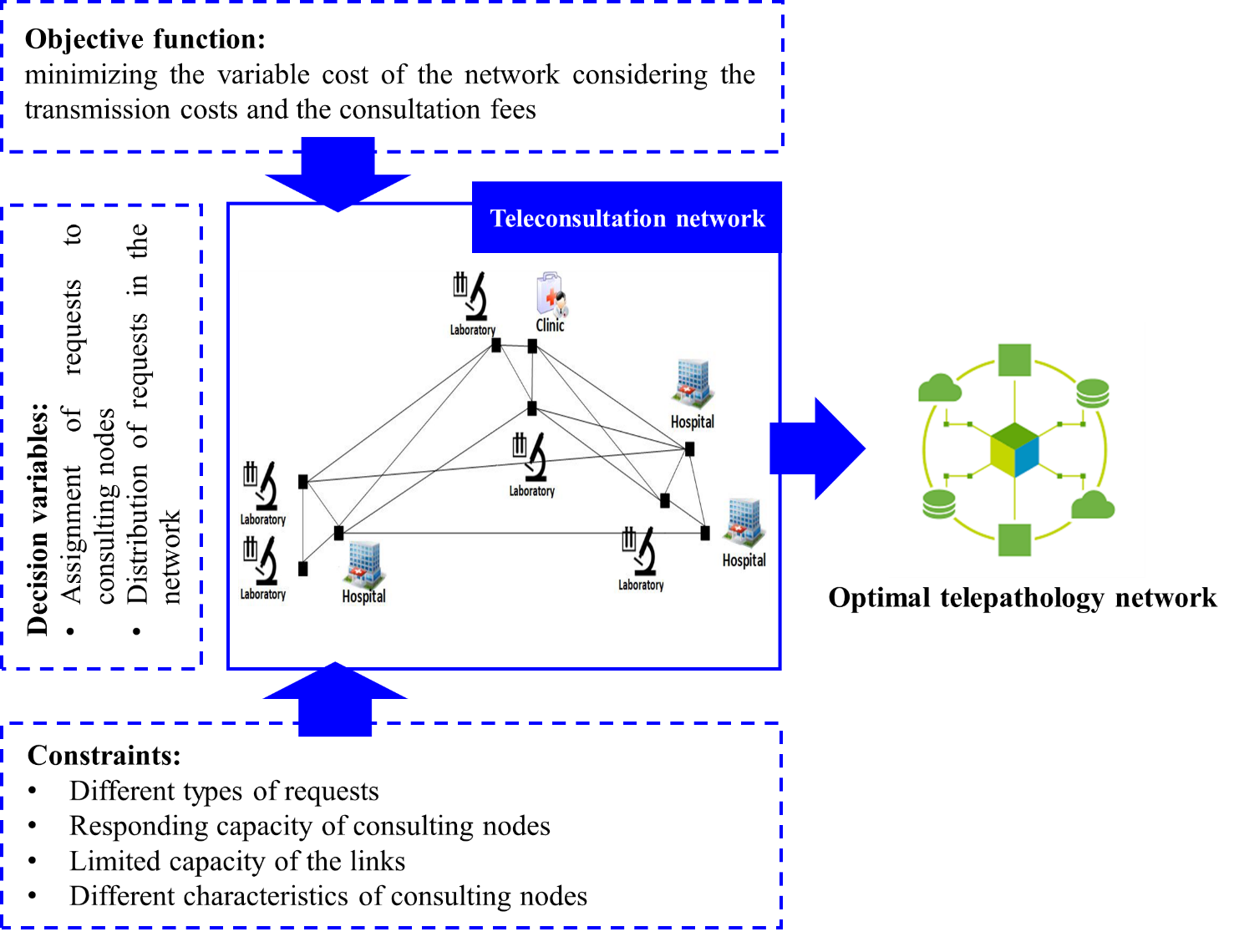

Supplement: Supplementary file 1 — Additional file 1: Fig. 1 The framework of the proposed methodology [file 12911_2020_1170_MOESM1_ESM.docx]

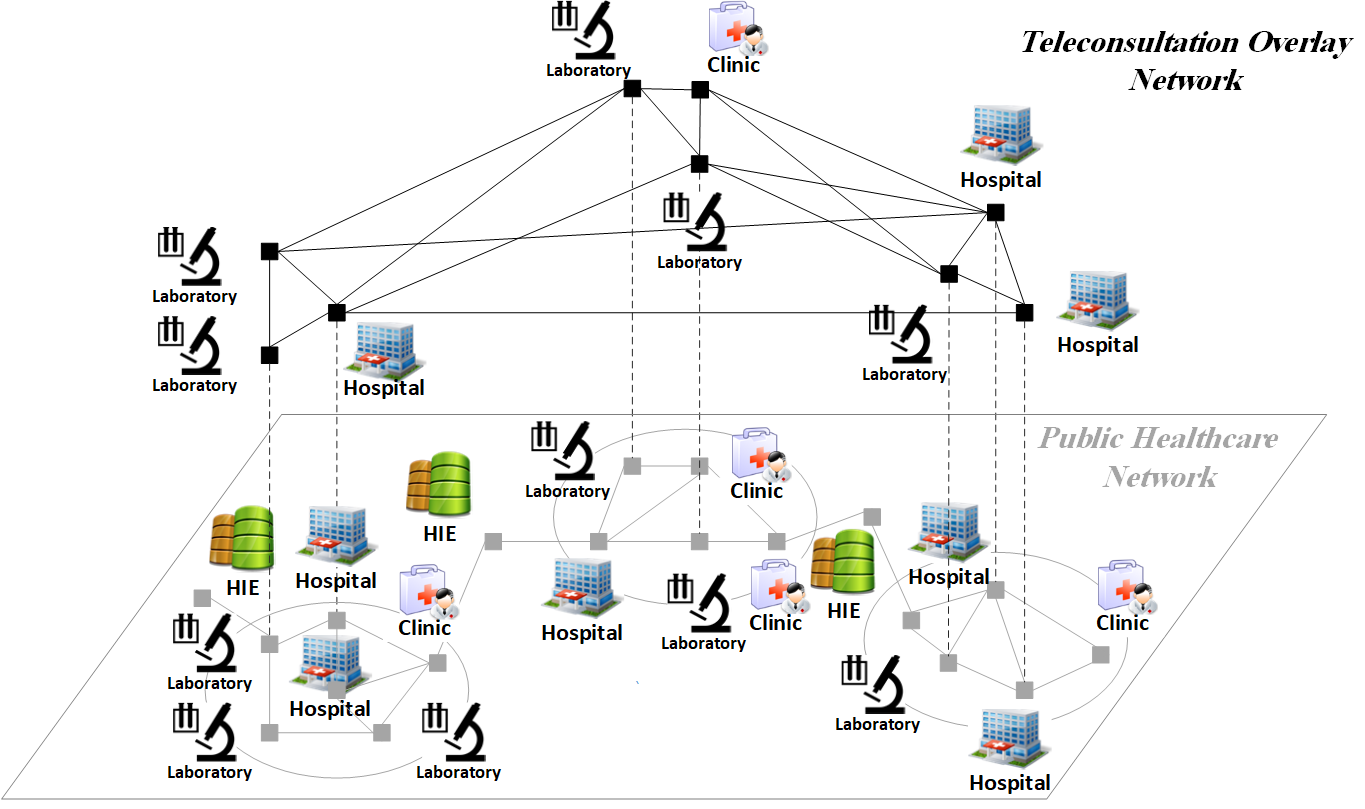

Supplement: Supplementary file 2 — Additional file 2: Fig. 2 An overlay architecture for healthcare teleconsultation networks. [file 12911_2020_1170_MOESM2_ESM.png]

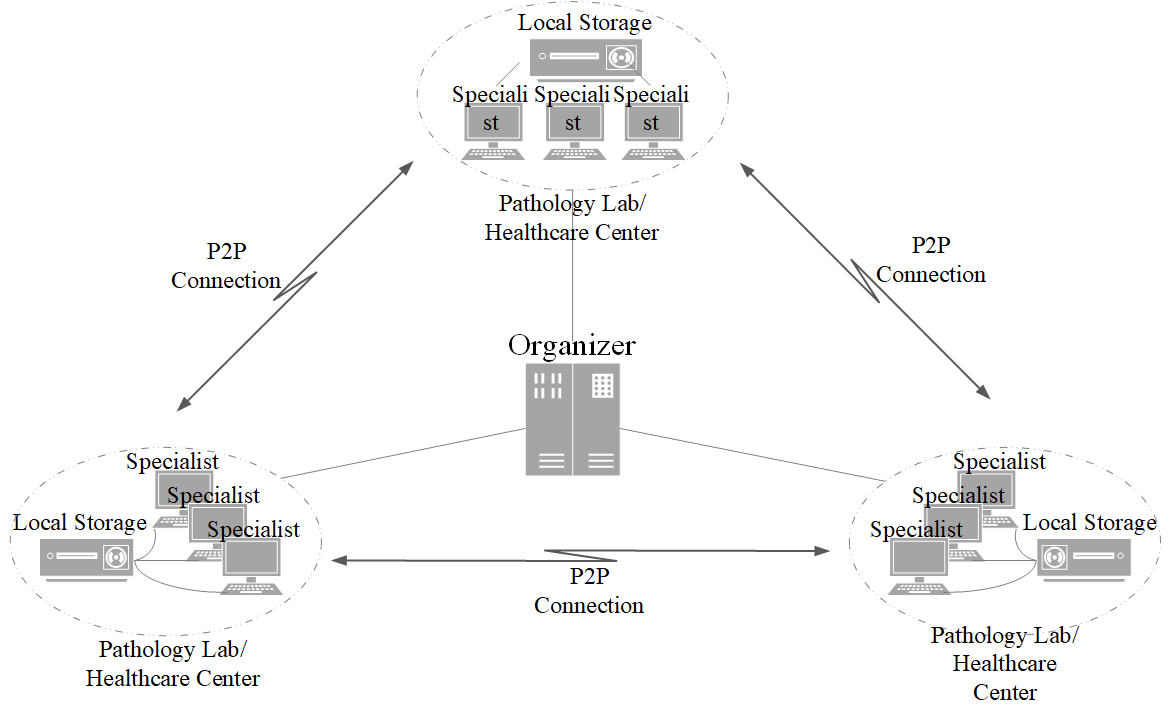

Supplement: Supplementary file 4 — Additional file 4: Fig. 4 The proposed hybrid architecture for healthcare teleconsultation networks [file 12911_2020_1170_MOESM4_ESM.png]

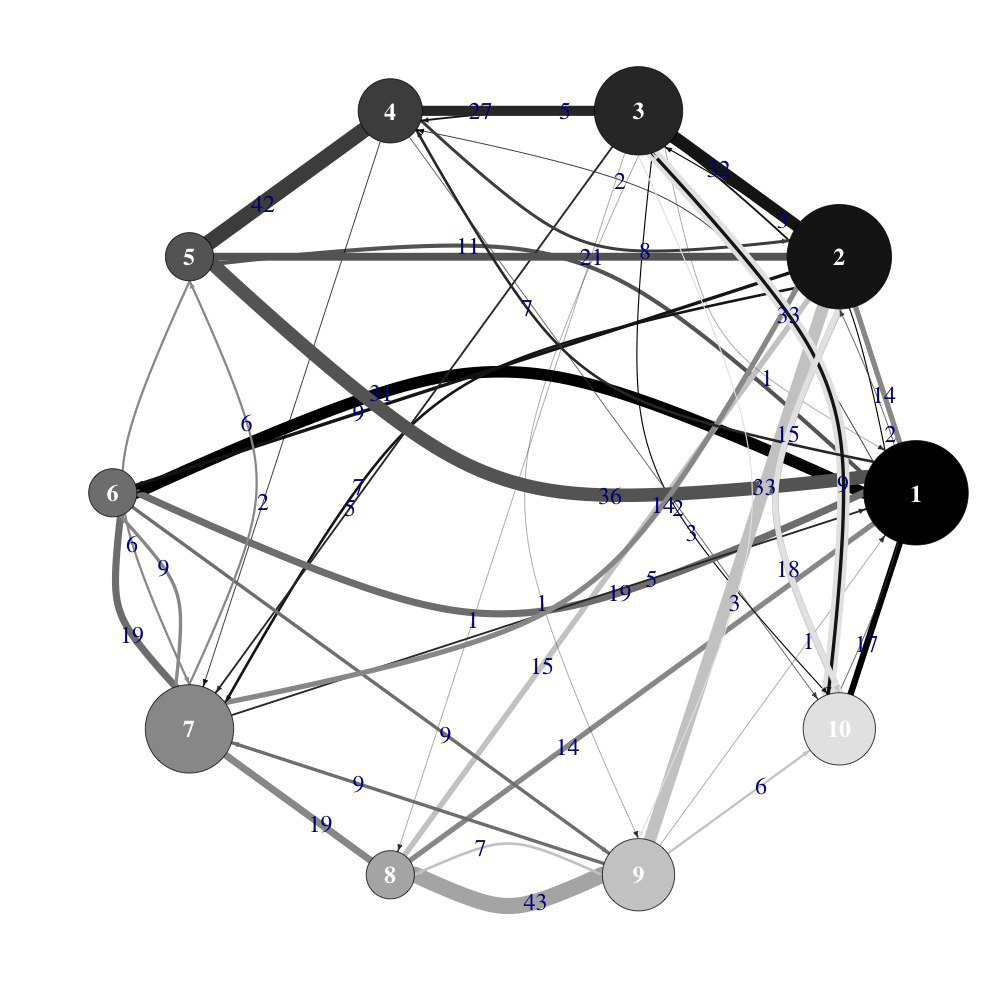

Supplement: Supplementary file 5 — Additional file 5: Fig. 5 A visualized solution for a network of 10 nodes, nodes and links with a bigger flow have a bigger size [file 12911_2020_1170_MOESM5_ESM.png]

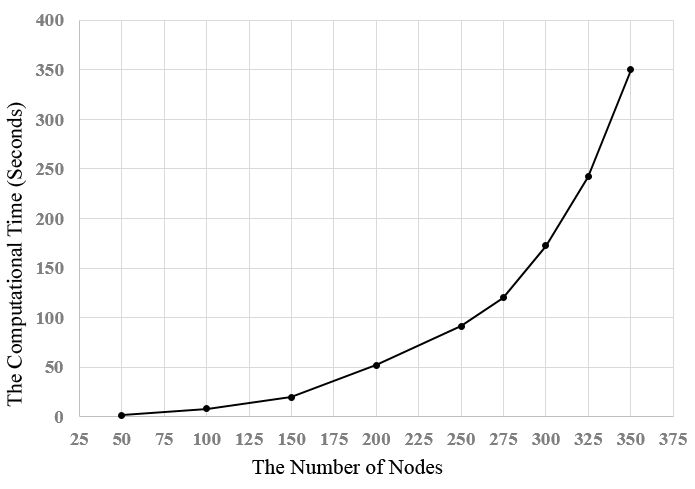

Supplement: Supplementary file 6 — Additional file 6: Fig. 6 The computational time versus the number of nodes [file 12911_2020_1170_MOESM6_ESM.png]

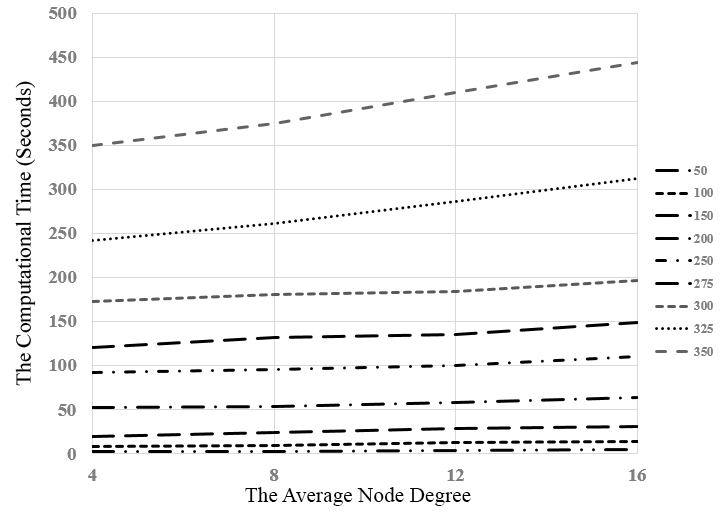

Supplement: Supplementary file 7 — Additional file 7: Fig. 7 The computational time versus the average node degree [file 12911_2020_1170_MOESM7_ESM.png]

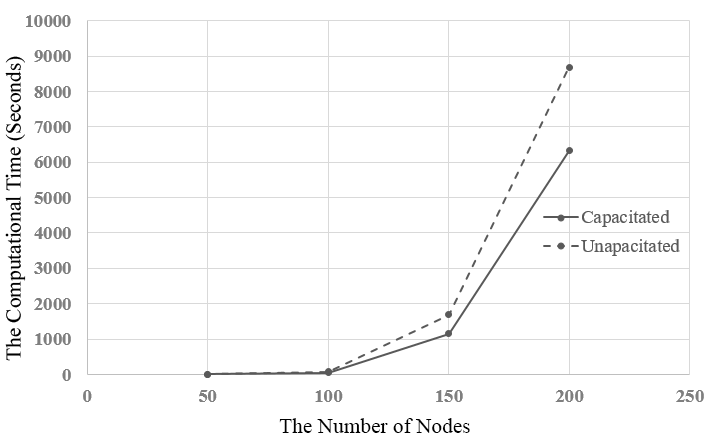

Supplement: Supplementary file 8 — Additional file 8: Fig. 8 The computational time for the capacitated and uncapacitated networks [file 12911_2020_1170_MOESM8_ESM.png]

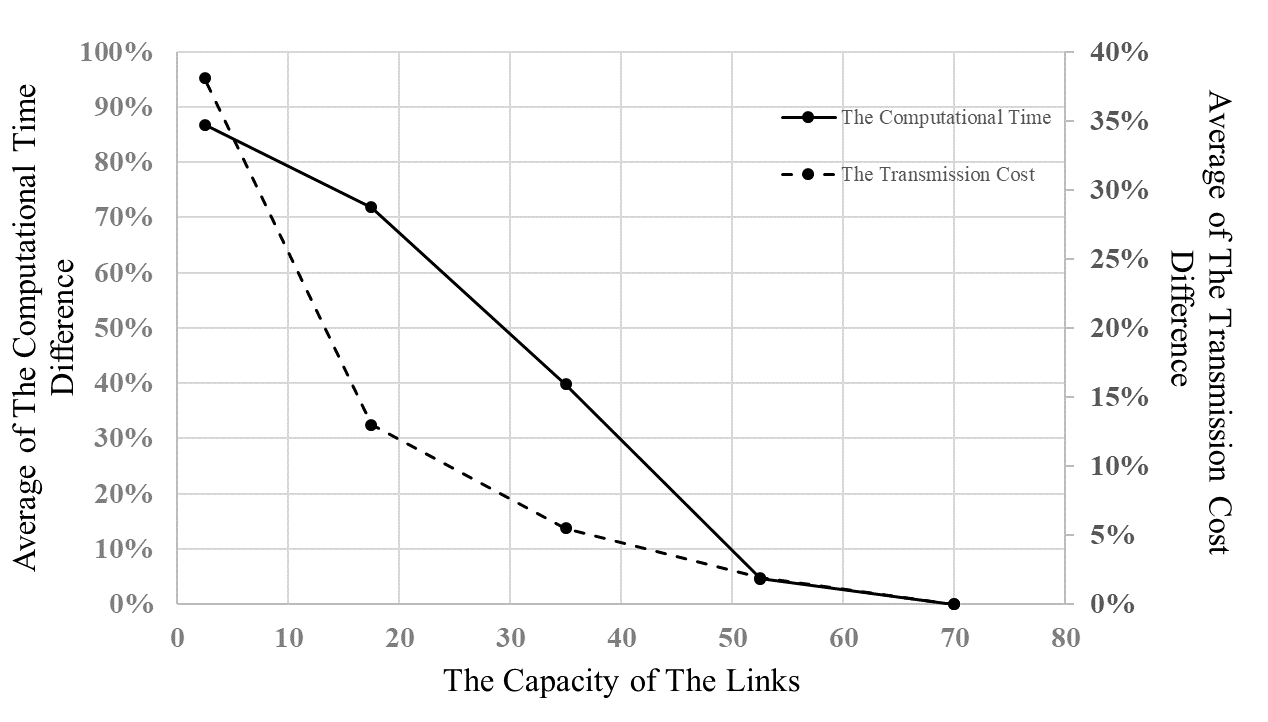

Supplement: Supplementary file 9 — Additional file 9: Fig. 9 Average of the computational time and the transmission cost differences versus the capacity of the links [file 12911_2020_1170_MOESM9_ESM.png]
