# Supplementary material for: Designing a novel hybrid healthcare teleconsultation network: a benchtop study of telepathology in Iran and a systematic review
Source: BMC Med Inform Decis Mak. 2020 Aug 12;20:186. doi: 10.1186/s12911-020-01170-6 (PMC7477836; doi:10.1186/s12911-020-01170-6)
Supplement: Supplementary file 3 — Additional file 3: Fig. 3 The PRISMA flow diagram of the literature search [file 12911_2020_1170_MOESM3_ESM.doc]

**Screening**

**Included**

**Eligibility**

**Identification**

**Records identified through database searching**
(n = 637)

**Additional records identified through other sources** (Reference books)**:**

(n =5)

**Records after duplicates removed**

(n =329)

**Records screened**
(n =329)

**Records excluded** (n =264)

Reports of Telepathology Systems=52

Studies of Optimization Problems in P2P Overlay Networks=193

**Full-text articles assessed for eligibility**
(n =84)

**Full-text articles excluded, with reasons** (n =53)

Reports of Telepathology Systems =10

- Not up-to-date articles
- Irrelevant information
- Conceptual Model

Studies of Optimization Problems in P2P Overlay Networks=43

- Different methodology
- Different assumptions
- Different architecture
- Different objective function

**Studies included in qualitative synthesis**
(n = 31)

**Reports of Telepathology Systems**

(n = 21)

**Studies of Optimization Problems in P2P Overlay Networks**

(n =10)
