# Supplementary material for: Designing a novel hybrid healthcare teleconsultation network: a benchtop study of telepathology in Iran and a systematic review
Source: BMC Med Inform Decis Mak. 2020 Aug 12;20:186. doi: 10.1186/s12911-020-01170-6 (PMC7477836; doi:10.1186/s12911-020-01170-6)
Supplement: Supplementary file 10 — Additional file 10: Appendix I. The Search Strategy [file 12911_2020_1170_MOESM10_ESM.docx]

Appendix I. The Search Strategy

.

# Search strategy

In this section, we present the search strategy used for Google Scholar. As we mentioned, our subject is an interdisciplinary area, and there are many studies related to this subject but from different perspectives. Therefore, we provided an overview of the academic publications mainly in two areas: 1) papers/reports of telepathology systems, and 2) optimization methods for request assignment and content distribution in P2P and overlay networks.

For each area, first, we started searching on Google Scholar with a few general keywords. Then, with the help of studies that seemed to be related to our work, we extracted our accurate keywords which are: medical consultation networks, teleconsultation, telepathology, P2P healthcare networks, peer to peer networks, overlay networks, resource planning, task assignment, content distribution, routing, mathematical model and optimization.

After that, since a full-text search on Google Scholar leads to thousands of irrelevant results, we used two Google advanced search operators (“intitle” and “intext”) in the following queries:

- intitle:[ "consultation" AND "network" OR "system"] intext:["medical consultation" AND "best practice" OR "resource planning" OR "mathematical model" OR "optimization" ]
- intitle:[ "teleconsultation" AND "network" OR "system"] intext:["medical consultation" AND "best practice" OR "resource planning" OR "mathematical model" OR "optimization" ]
- intitle:[ "consultation" AND "network" OR "system"] intext:["pathology" AND "best practice" OR "resource planning" OR "mathematical model" OR "optimization" ]
- intitle:[ "consultation" AND "network" OR "system"] intext:["telepathology" AND "best practice" OR "resource planning" OR "mathematical model" OR "optimization" ]
- intitle:[ "consultation" AND "network" OR "system"] intext:["telemedicine" AND "best practice" OR "resource planning" OR "mathematical modeling" OR "optimization" ]
- intitle:[ "peer to peer" AND "healthcare" AND "system" OR "network"] intext:["consultation" OR "optimization" OR "resource planning"]
- intitle:[ "p2p" AND "healthcare" AND "system" OR "network"] intext:["consultation" OR "optimization" OR "resource planning"]
- intitle:[ "overlay" AND "healthcare" AND "system" OR "network"] intext:["consultation" OR "optimization" OR "resource planning"]
- intitle:[ "p2p" AND "task assignment" OR "Content distribution"] intext:[ "mathematical model" AND "optimization" OR "resource planning" OR "overlay"]
- intitle:[ "peer to peer" AND "task assignment" OR "Content distribution"] intext:[ "mathematical model" AND "optimization" OR "resource planning" OR "overlay"]
- intitle:[ "p2p" AND "routing" AND "assignment" ] intext:[ "optimization" AND "mathematical model"]
- intitle:[ "peer to peer" AND "routing" AND "assignment" ] intext:[ "optimization" AND "mathematical model"]
- intitle:[ "p2p" AND "distribution" AND "assignment" ] intext:[ "optimization" AND "mathematical model"]
- intitle:[ "peer to peer" AND "distribution" AND "assignment" ] intext:[ "optimization" AND "mathematical model"]
